# Perceptual Decisions in the Presence of Relevant and Irrelevant Sensory Evidence

**DOI:** 10.3389/fnins.2017.00618

**Published:** 2017-11-10

**Authors:** Ursula M. Anders, Charlotte S. McLean, Bowen Ouyang, Jochen Ditterich

**Affiliations:** Center for Neuroscience and Department of Neurobiology, Physiology and Behavior, University of California, Davis, Davis, CA, United States

**Keywords:** computational model, gain modulation, irrelevant sensory evidence, linear combination, perceptual decision, relevant sensory evidence

## Abstract

Perceptual decisions in the presence of decision-irrelevant sensory information require a selection of decision-relevant sensory evidence. To characterize the mechanism that is responsible for separating decision-relevant from irrelevant sensory information we asked human subjects to make judgments about one of two simultaneously present motion components in a random dot stimulus. Subjects were able to ignore the decision-irrelevant component to a large degree, but their decisions were still influenced by the irrelevant sensory information. Computational modeling revealed that this influence was not simply the consequence of subjects forgetting at times which stimulus component they had been instructed to base their decision on. Instead, residual irrelevant information always seems to be leaking through, and the decision process is captured by a net sensory evidence signal being accumulated to a decision threshold. This net sensory evidence is a linear combination of decision-relevant and irrelevant sensory information. The selection process is therefore well-described by a strong linear gain modulation, which, in our experiment, resulted in the relevant sensory evidence having at least 10 times more impact on the decision than the irrelevant evidence.

## Introduction

Perceptual decision-making is the process of making a discrete choice based on available sensory information. Most studies of the underlying mechanisms have used paradigms where a decision-relevant sensory stimulus is presented in isolation. Perceptual decisions in everyday situations, however, usually have to be made in the presence of sensory stimuli that are not relevant to the decision at hand. Successful perceptual decisions therefore require a separation of decision-relevant from irrelevant sensory information. If decision-relevant and irrelevant information are present at different spatial locations, visuospatial attention can be allocated appropriately to preferentially process the relevant stimulus (Sapir et al., 2005; Wilimzig et al., 2008; Zizlsperger et al., 2012; Wyart et al., 2015). If decision-relevant and irrelevant information are present at the same spatial location, other selection mechanisms need to be at play. Feature-based attention could contribute in this case (Treue and Martinez Trujillo, 1999; Martinez-Trujillo and Treue, 2004; Maunsell and Treue, 2006), but we will discuss later that feature-based attentional modulation of sensory representations alone is unlikely to provide sufficient separation.

Several recent animal studies were targeted at investigating the neural mechanisms underlying this selection process. The experimental tasks tend to be modified versions of the well-known random-dot motion direction discrimination task, which has been very influential in uncovering mechanisms of perceptual decision-making (Shadlen and Newsome, 2001; Roitman and Shadlen, 2002; Palmer et al., 2005). Sasaki and Uka used a random-dot motion stimulus that also contained depth information, and monkeys had to perform either a motion direction or depth discrimination (Sasaki and Uka, 2009). Other studies used a colored random-dot motion stimulus, and monkeys had to perform either a motion direction or color discrimination (Mante et al., 2013; Siegel et al., 2015). Sasaki and Uka recorded from the middle temporal area and found the representation of sensory evidence unaffected by the decision relevance of a particular stimulus feature (Sasaki and Uka, 2009). Mante et al. proposed that monkeys solve the task by taking advantage of a selective integration mechanism, and neural activity recorded from prefrontal cortex seemed consistent with such a mechanism (Mante et al., 2013). Siegel et al. recorded neural activity from a larger network of cortical areas, which suggested a more distributed decision-making process (Siegel et al., 2015).

Here we use human decision behavior and computational modeling to gain insight into the decision process. We used a motion-only version of the task, which has the advantage that decision-relevant and irrelevant information are represented by sensory neurons with the same properties, and quantifying the neural representation of the sensory evidence is therefore relatively straightforward, as neural responses to random-dot motion stimuli have been studied in detail (Britten et al., 1993). Subjects saw a two-component random-dot stimulus, which contained both horizontal and vertical motion. Only one of these components was decision-relevant on any given trial and subjects had to perform either a horizontal (left vs. right) or a vertical motion discrimination (up vs. down). We had first used this type of stimulus in an fMRI study on cognitive control signals associated with the different types of conflicts that arise when performing this task (Wendelken et al., 2009). In this earlier study, only two different motion strengths (coherence levels) were used and decision times were not measured. Choices were well-captured by a logistic function of a linear combination of the decision-relevant and irrelevant coherences, suggesting that the selection process might be described as a linear gain modulation of the sensory evidence. Here we demonstrate that this model of the decision process can not only capture choices over a wide range of difficulty levels, but also account for the amount of time that subjects require to make up their mind when performing this task. We further show that incomplete suppression of the decision-irrelevant sensory information is required to explain the decision behavior and that lapses in remembering which stimulus component had been cued alone are insufficient to account for the observed behavior.

## Materials and methods

### Human subjects

Thirteen UC Davis undergraduate students, both male and female, participated in the study. This study was carried out in accordance with the recommendations of the U.S. Department of Health and Human Services regulations for the protection of human subjects in research with written informed consent from all subjects. All subjects gave written informed consent in accordance with the Declaration of Helsinki. The protocol was approved by the UC Davis Institutional Review Board. All subjects had normal or corrected to normal vision. Each subject completed at least five experimental sessions, resulting in a minimum of 1,500 experimental trials per subject. Since the maximum likelihood approach that was used for computational modeling requires a substantial number of trials for each experimental condition, we pooled data across subjects, which requires decision behavior to be comparable across all the subjects within the pool. We therefore initially screened the data for potential outliers. For each subject, we determined the mean response time (RT) for each experimental condition and took the minimum mean RT as a measure of how quickly each subject responded in an experimental condition leading to fast decisions. Cluster analysis [k-means clustering using the Davies-Bouldin criterion (Davies and Bouldin, 1979) and verified by silhouettes (Rousseeuw, 1987)] of this value across subjects revealed two clusters: two subjects were substantially slower than everybody else. We excluded these two subjects from the data pool as inclusion would have resulted in bimodal RT distributions. To further verify that the pooling did not create RT distributions that are inconsistent with those of individual subjects we performed a group reaction time distribution analysis as suggested in Ratcliff (1979). We determined the quantiles of individual subjects' RT distributions and compared their average (“group RT distribution”) to the quantiles of the pooled RT distributions. The comparison (Supplementary Figure 1) revealed that the pooled RT distributions were similar to the group RT distributions, only slightly wider, which the computational models that are used in this paper can easily absorb as slightly increased variability in the residual time (see Model of the decision process). The second parameter that we analyzed was the influence of the irrelevant motion component on the choice behavior (see Data Analysis for how this influence was quantified). Typically, subjects would show an increased likelihood of choosing the target associated with the irrelevant direction of motion (positive slope of the psychometric function when analyzing trials with only irrelevant coherent motion). Only one subject showed the opposite effect (negative slope). This subject was excluded from the data pool to ensure comparable choice behavior. Our analyses in this paper are therefore based on the pooled data from 10 subjects. This data pool contained 14,218 valid (see Experimental Task and Visual Stimulus) decision trials.

### Experimental setup

The subjects sat in front of a 22″ flat-screen CRT video monitor (ViewSonic P225f; viewing distance: 60 cm) with their head on a chin and forehead rest. The visual stimuli were generated by a Macintosh G4 computer running Mac OS 9, MATLAB (The Mathworks, Natick, MA), and the Psychophysics Toolbox (Brainard, 1997; Pelli, 1997) at a frame rate of 75 Hz. The experiment was controlled and the data were collected by an Intel Pentium IV computer running QNX (Ottawa, ON, Canada) and a modified version of REX (Laboratory of Sensorimotor Research, National Eye Institute). Eye movements were monitored using an IR video eye tracker (Model 5,000, Applied Science Laboratories, Bedford, MA). The eye position of one eye was sampled at 240 Hz. Prior to each experimental session the eye tracker was calibrated using repeated fixation of nine calibration targets.

### Experimental task and visual stimulus

**Figure 1:**
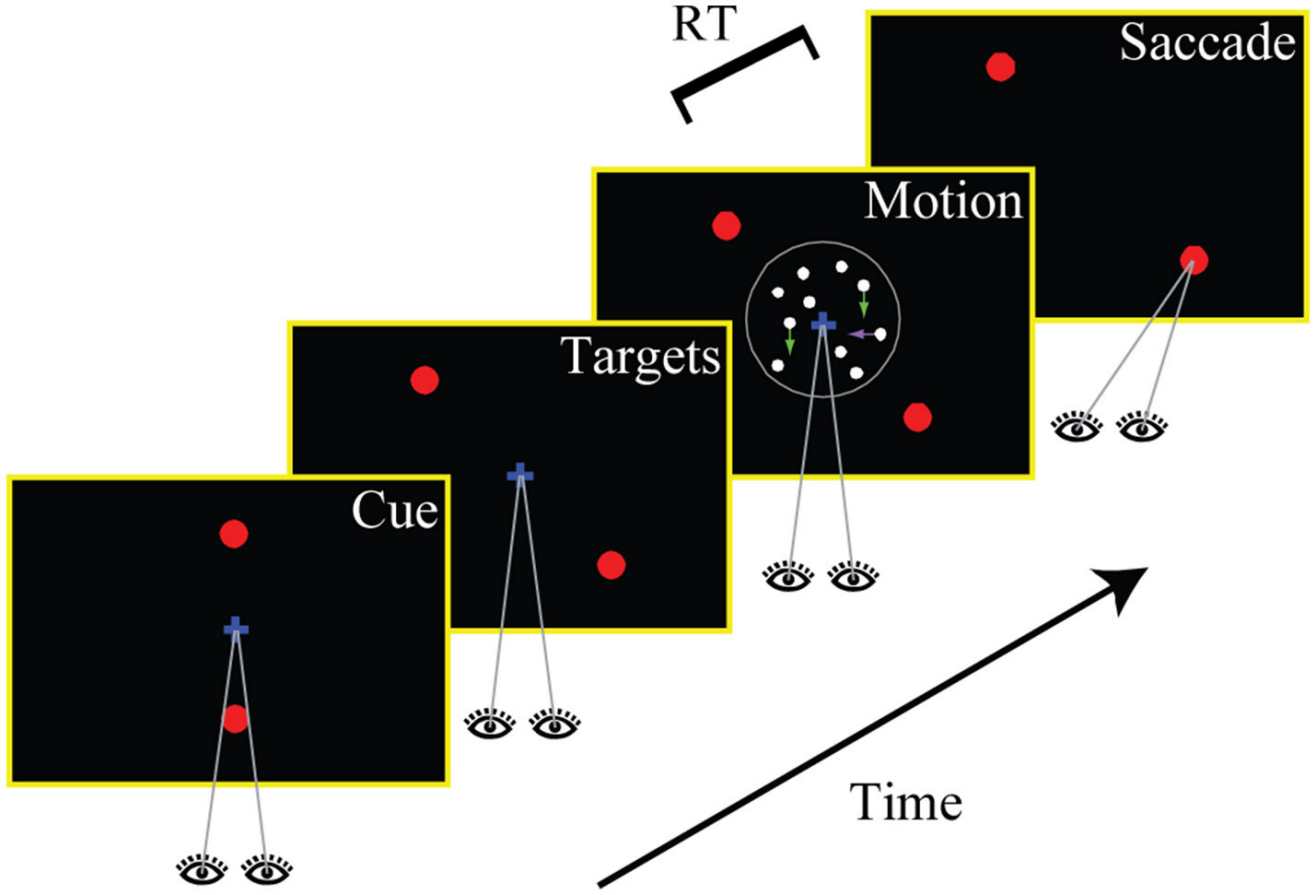
Experimental task. Subjects made perceptual decisions about the direction of motion in a random dot stimulus that contained both horizontal and vertical coherent motion. Only one of the two components was decision-relevant on any given trial (indicated by a cue at the beginning of the trial; the vertical axis is cued in the shown example). Two choice targets appeared along one of the two possible diagonals, and subjects were instructed to make a goal-directed eye movement to the target closest to the identified relevant direction of motion. The stimulus viewing duration was controlled by the subjects and choices and response times were measured.

The experimental task is illustrated in Figure 1. Each trial started with the presentation of a central fixation mark (diameter: 0.3°). The measured fixation location had to remain within 2.5° of the center of the screen throughout the trial (up to the saccadic response). After 500 ms of stable fixation, two targets (diameter: 0.5°) appeared on the screen, either 5.7° to the left and to the right of the center (horizontal cue) or 5.7° above and below the center (vertical cue). The cue was chosen randomly with equal probability on every single trial and served as an instruction to the subject whether a decision about horizontal or vertical motion had to be made on the given trial. The cue remained on the screen for 750 ms, after which the targets jumped to new locations along one of the two diagonals (again randomly chosen) with eccentricities of 8.0° (either bottom left and top right, or top left and bottom right). After a random delay (min.: 0.3 s, max.: 1.0 s, truncated exponential distribution), a multi-component random-dot pattern was presented at the center of the screen (diameter: 5.0°).

In the original version of the stimulus (as used, e.g., in Shadlen and Newsome, 2001; Roitman and Shadlen, 2002; Palmer et al., 2005) a certain fraction of the dots (defined as the coherence of the stimulus) was moving coherently in a particular direction whereas the rest of the dots were flickering randomly. Our multi-component random-dot pattern had up to two coherent motion components embedded (for a previous use of this type of stimulus with three coherent components see Niwa and Ditterich, 2008). Thus, in our case, there were three subpopulations of dots: one of them was moving coherently either to the left or to the right (fraction of dots defined by the coherence of the horizontal component), one was moving coherently either up or down (fraction of dots defined by the coherence of the vertical component), and the rest of the dots were flickering randomly. The stimulus is therefore described by a set of two coherences. Which of the three subpopulations a particular dot belonged to changed randomly over the course of the stimulus. As a consequence, the stimulus is not perceived as an overlay of several transparent layers of motion that could be easily separated, but as a mixture of different motion components. See Treue et al. (2000) for a discussion of transparent random-dot motion stimuli. Corresponding pairs of dots, responsible for the percept of apparent motion, were presented with a temporal separation of 40 ms (3 video frames). The coherently moving dots had a speed of 6 deg/s, the dot density was $16.7\frac{dots}{\deg^{2}\cdot s}$, and each dot was a little filled square with an edge length of 0.1°. On each trial, the coherence of each component was randomly chosen to be either 0, 10, 20, or 30%, the horizontal direction was randomly chosen to be either left of right, and the vertical direction was randomly chosen to be either up or down. Some of the subjects also saw additional coherence combinations, but we limit our analysis here to these combinations, which were experienced by all subjects.

The subjects were instructed to identify the direction of the relevant (cued) motion component, to ignore motion along the irrelevant axis, and to make a saccadic eye movement to the one describes the responseassociated choice target (closest to the identified direction of motion). Each choice target was therefore associated with one horizontal direction and one vertical direction. The two motion components could either provide evidence for the same target (we will refer to these types of trials as “congruent” trials) or for opposite targets (“incongruent” trials). Subjects were allowed to watch the stimulus for as long as they wanted to (up to 5 s) and responded whenever they were ready. After each trial they received auditory feedback as to whether they had picked the correct target. The computer always identified the target associated with the direction of motion along the cued axis as the correct one. Since such a direction was also randomly determined in the case of stimuli without relevant coherent motion, one of the targets was randomly chosen to be the correct one.

In order to complete a trial successfully (“valid trial”), the subject had to maintain accurate fixation until the random-dot pattern appeared. Once central fixation was broken, the eye position had to be within 3.0° of one of the two choice targets within 100 ms and had to stay on this target for at least 200 ms.

### Data analysis

When analyzing the data, we collapsed across different target locations and whether the horizontal or the vertical motion axis was cued as relevant. Thus, we only considered the set of coherences, whether the irrelevant component provided evidence for the same (“congruent” trial) or for the opposite target (“incongruent” trial) as the relevant component, and whether the subject picked the target associated with the direction of the relevant motion component (“correct” choice) or not (“error”). The motion strength of the irrelevant component was treated as a positive value when the associated direction provided evidence for choosing the correct target (“congruent trials”) and as a negative value when the opposite target was supported (“incongruent trials”).

When analyzing choice data from the random-dot motion direction discrimination task, the psychometric function is usually well-described by a logistic function of the form $p\left(correct\;choice\right)=\frac{e^{\alpha \cdot c}}{1+e^{\alpha \cdot c}}$ (Roitman and Shadlen, 2002; Palmer et al., 2005), with *c* being the coherence of the stimulus and α defining the slope of the psychometric function. We used the same function to capture how choices were influenced by coherent motion when only one of the stimulus components had non-zero strength. The slope parameter α was determined using maximum likelihood estimation. The standard error of the parameter was calculated from the second partial derivative of the log likelihood with respect to the parameter (Meeker and Escobar, 1998). For stimuli with both motion components having non-zero coherence we evaluated a function of the form

$$\begin{array}{l} p\left(correct\;choice\right)\;=\;\frac{e^{\left(\alpha_{rel}\cdot c_{rel}\;+\;\alpha_{irr}\cdot c_{irr}\right)}}{1+e^{\left(\alpha_{rel}\cdot c_{rel}\;+\;\alpha_{irr}\cdot c_{irr}\right)}} \end{array}$$

based on the idea that choices could be made on the basis of a combination of relevant and irrelevant sensory evidence. *c*_*rel*_ is the strength of the relevant motion component, *c*_*irr*_ is the strength of the irrelevant component, with a negative value indicating an incongruent trial, and α_*rel*_ and α_*irr*_ describe how much impact the relevant and the irrelevant sensory evidence have on the choice. In the case of only one motion component with non-zero coherence this function reduces to the standard logistic function mentioned above.

The response time (RT) was measured as the time between the appearance of the random-dot stimulus and the breaking of central fixation.

### Computational model

A general description of the ideas behind our model can be found in the Results section.

#### Model of the neural representation of the sensory stimulus

Since we used the same type of multi-component random-dot motion stimulus as in an earlier study (Niwa and Ditterich, 2008) we took a very similar approach to modeling the neural representation of the sensory stimulus. Specifically, the mean response of a population of motion-sensitive neurons to a two-component random-dot stimulus with coherences *c*_1_ (in the preferred direction of the pool; 0 ≤ *c*_1_ ≤ 1) and *c*_2_ (orthogonal to the preferred direction; 0 ≤ *c*_2_ ≤ 1) was modeled to be of the form

$$\begin{array}{l} \bar{s_{1}}=\frac{g\cdot \left[c_{1}+k_{n}\cdot \left(1-\overset{2}{\underset{i=1}{\sum }}c_{i}\right)\right]}{1+k_{s}\cdot \overset{2}{\underset{i=1}{\sum }}c_{i}} \end{array}$$

where *g* is the overall gain of the sensory response (relationship between neural activity and motion strength). The two additive terms in the brackets reflect the two linear response components: the first one describes the response to the coherent motion in the preferred direction, the second one describes the response to the noise dots. The term in parenthesis reflects the proportion of noise dots in the stimulus. *k*_*n*_ is the relative gain of the response to the noise dots compared to the response to an identical fraction of dots moving coherently in the preferred direction. The term in the denominator reflects the divisive normalization. For simplicity, we have chosen a linear term with *k*_*s*_ describing the gain/strength of the divisive normalization. The sum following *k*_*s*_ reflects the total amount of coherent motion in the stimulus and is therefore a proxy for the overall activation of the population across preferred directions. This approximation turns out to be sufficient to capture the structure of the behavioral data.

In general, the mean responses of each of the four task-relevant sensory pools (tuned to leftward, rightward, upward, and downward motion) can be written as

$$\begin{array}{l} \bar{s_{dir}}=\frac{g\cdot \left[c_{dir}+k_{n}\cdot \left(1-c_{tot}\right)\right]}{1+k_{s}\cdot c_{tot}} \end{array}$$

with *c*_*dir*_ being the coherence of motion in the preferred direction of the pool (0 if there is coherent motion in the opposite direction) and *c*_*tot*_ = *c*_*rel*_ + |*c*_*irr*_| being the total amount of coherent motion in the stimulus.

The variances of the four sensory responses were modeled as

$$\begin{array}{l} \sigma_{s_{dir}}^{2}=k_{v}\cdot \bar{s_{dir}} \end{array}$$

As the sensory evidence has to be mediated by pools of spiking neurons with Poisson-like properties, it is reasonable to assume that the variability of the signal potentially increases with its mean (see Ditterich, 2006 for a more detailed discussion of this topic). The proportionality factor *k*_*v*_ was determined empirically through the model fit.

We described the outputs of the sensory pools as normal random processes to be able to treat the decision process as a standard diffusion process (based on Brownian motion), which is reasonable if the pools are not too small.

The mean sensory response does not include baseline activity, which would subtract out again at the opponent readout stage (see next section). It could make a contribution to the variability of the sensory response though, but we allow for a stimulus-independent noise component of the decision signal (see below), which can also include contributions due to baseline activity of sensory neurons.

#### Model of the decision process

Similar to previous work on perceptual decision-making involving the random-dot motion direction discrimination task (Palmer et al., 2005; Ditterich, 2006; Niwa and Ditterich, 2008), but also other tasks, we assume that decisions are based on accumulating sensory evidence and comparing accumulated evidence to a decision threshold, which can be formalized as a bounded drift-diffusion process (Ratcliff and McKoon, 2008). Sensory activity for opposing directions is initially subtracted to obtain the net sensory evidence for, e.g., leftward vs. rightward motion. The net sensory evidence then feeds into a neural integrator, which accumulates the net sensory evidence over time. As soon as the accumulated evidence exceeds a decision threshold the decision terminates, which determines the decision time, and the bound that is hit first determines which choice is made.

In our case, subjects are making a choice between two possible targets, but there is decision-relevant and irrelevant net sensory evidence. Our model assumes that the separation of the two components is potentially not perfect and that the decision is informed by a linear combination of both signals, with the irrelevant information being weighed less (*k*_*irr*_ < 1). **Figure 3** shows the structure of the decision mechanism, illustrated for the example of targets appearing in the upper-left and lower-right corners and the vertical motion axis being cued (as in Figure 1). Let's further assume that the coherent vertical motion is downward. The top integrator collects evidence for picking the lower right target, which would be the correct choice in the given example, the bottom integrator collects evidence for picking the upper left target. The situation shown in the diagram, two integrators racing against each other for a threshold crossing, is equivalent to a single integrator with two decision bounds, one above and one below the starting point of integration, if the signals feeding into the two integrators are perfectly anti-correlated, which is the case in our model (*e*_2_ = −*e*_1_). One parameter can always be picked arbitrarily in a bounded drift-diffusion model and we decided to place the decision bounds of the one-dimensional drift-diffusion process at +1 and −1, with the accumulation process starting at 0. The evidence signal that is accumulated in support of picking the correct target (lower right in this case) is

$$\begin{array}{l} e_{1}=\left(s_{down}-s_{up}\right)+k_{irr}\cdot \left(s_{right}-s_{left}\right)+n \end{array}$$

with n allowing for some additional stimulus-independent, zero-mean noise on the evidence signal. This signal that feeds into the integrator has a mean of

$$\begin{array}{l} \bar{e_{1}}=\left(\bar{s_{down}}-\bar{s_{up}}\right)+k_{irr}\cdot \left(\bar{s_{right}}-\bar{s_{left}}\right) \end{array}$$

which corresponds to the drift rate of the drift-diffusion process and a variance of

$$\begin{array}{l} \sigma_{e_{1}}^{2}=\left(\sigma_{s_{down}}^{2}+\sigma_{s_{up}}^{2}\right)+k_{irr}^{2}\cdot \left(\sigma_{s_{right}}^{2}+\sigma_{s_{left}}^{2}\right)+\sigma_{n}^{2} \end{array}$$

which corresponds to the diffusion component of the drift-diffusion process. In general, for any combination of target locations, cued axis, and coherences, this will be

$$\begin{array}{l} \bar{e_{1}}=\frac{g\cdot \left(c_{rel}+k_{irr}\cdot c_{irr}\right)}{1+k_{s}\cdot c_{tot}} \end{array}$$

with the upper bound corresponding to a correct choice and

$$\begin{array}{l} \sigma_{e_{1}}^{2}=\frac{k_{v}\cdot g}{1+k_{s}\cdot c_{tot}}\cdot [c_{rel}+k_{irr}^{2}\cdot \left|c_{irr}\right|\\ +\;2k_{n}\cdot \left(1-c_{tot}\right)\;\cdot \;\left(1+k_{irr}^{2}\right)]+\sigma_{n}^{2} \end{array}$$

A correct choice will be made when *i*_1_ > +1 and an error (incorrect choice) will occur when *i*_1_ < −1.

The drift rate was allowed to vary randomly from trial to trial and was drawn from a normal distribution with mean $\bar{e_{1}}$ and standard deviation σ_*DR*_. This feature enables the drift-diffusion model to account for differences in correct and error response times (Ratcliff and Rouder, 1998).

For calculating the predictions of the model (probabilities of making a correct choice/error and decision time distributions for each coherence combination) we took advantage of a numerical solution to the first passage time problem (see Ditterich, 2006, Appendix B.1 and Smith, 2000 for details). The MATLAB function WIENER_VD_1D_2B_NUM.M, which has been used for performing the model calculations, is part of the Stochastic Integration Modeling Toolbox (written by JD), which can be downloaded from the Software section of http://www.peractionlab.org.

Response times were assumed to have two additive components: the decision time and a residual time, which could vary randomly from trial to trial and was assumed to be normally distributed. (Predicted RT distributions were obtained by convolving the decision time distributions with a Gaussian kernel.)

#### Model fit and predictions

Model parameters were identified by maximum likelihood estimation (using the combination of choice and RT). The log likelihood was obtained by summing, across all trials, the logs of the probabilities of obtaining each trial's particular combination of choice and RT according to the model:

$$\begin{array}{l} Log\;likelihood=\underset{all\;trials}{\sum }\ln\;P_{\text{model}}\left(choice,\;RT\right) \end{array}$$

A multi-dimensional simplex algorithm (provided by MATLAB's Optimization Toolbox) was used to find the combination of parameters that would maximize the likelihood of the experimental data. A pattern search (provided by MATLAB's Global Optimization Toolbox) was used to make sure that the simplex algorithm was not getting stuck in a local optimum. Predicted decision time distributions were calculated up to a minimum of 5,000 ms with a temporal resolution of 5 ms.

In some of our models (see main text for details) we allowed for the possibility that subjects might sometimes forget which motion axis had been cued by assuming that, on a certain proportion of the trials (*p*_*wrong*_*axis*_), the decision would be dominated by the irrelevant rather than the relevant component of the motion stimulus. The predicted behavior on these trials was obtained by exchanging the roles of the two motion components in the model: The irrelevant sensory information would receive a weight of 1, whereas the relevant information would only be weighted by *k*_*irr*_. The overall predicted behavior (choices and RT distributions) was then obtained as a weighted sum of (1− *p*_*wrong*_*axis*_) times the predicted behavior assuming appropriate use of the cue information and *p*_*wrong*_*axis*_ times the predicted behavior assuming that the irrelevant axis was given the larger weight.

For comparison purposes with the value estimated from the choice data, the slope of the model-predicted psychometric function for trials with only irrelevant coherent motion was determined by a least-squares fit of a logistic function:

$$\begin{array}{l} p\left(correct\;choice\right)=\frac{e^{\alpha_{model}\cdot c_{irr}}}{1+e^{\alpha_{model}\cdot c_{irr}}} \end{array}$$

The standard error of the estimated slope was determined using the principle of error propagation as typically done in non-linear regression:

$$\begin{array}{l} \Sigma_{p}={\left(J^{T}J\right)}^{-1}\cdot \sigma_{res}^{2} \end{array}$$

Σ_*p*_ is the parameter covariance matrix, in our case just the variance of the estimated slope, *J* is the Jacobian, and $\sigma_{res}^{2}$ the variance of the residuals. The two slopes (data-based α and model-based α_*model*_) were then compared using a *t*-test.

## Results

We asked human subjects to make a perceptual decision about the direction of motion in a random dot stimulus. Importantly, the stimuli contained two orthogonal components of coherent motion, but only one component was decision-relevant on any given trial. A cue at the beginning of each trial indicated whether a decision was supposed to be made about motion along the horizontal or the vertical axis. Two choice targets appeared along one of the two possible diagonals such that each target was associated with one horizontal and one vertical direction of motion. Subjects made a goal-directed saccade to one of the targets whenever ready, and we measured choices and response times (RT). Figure 1 shows the structure of the task.

### Decision behavior

**Figure 2:**
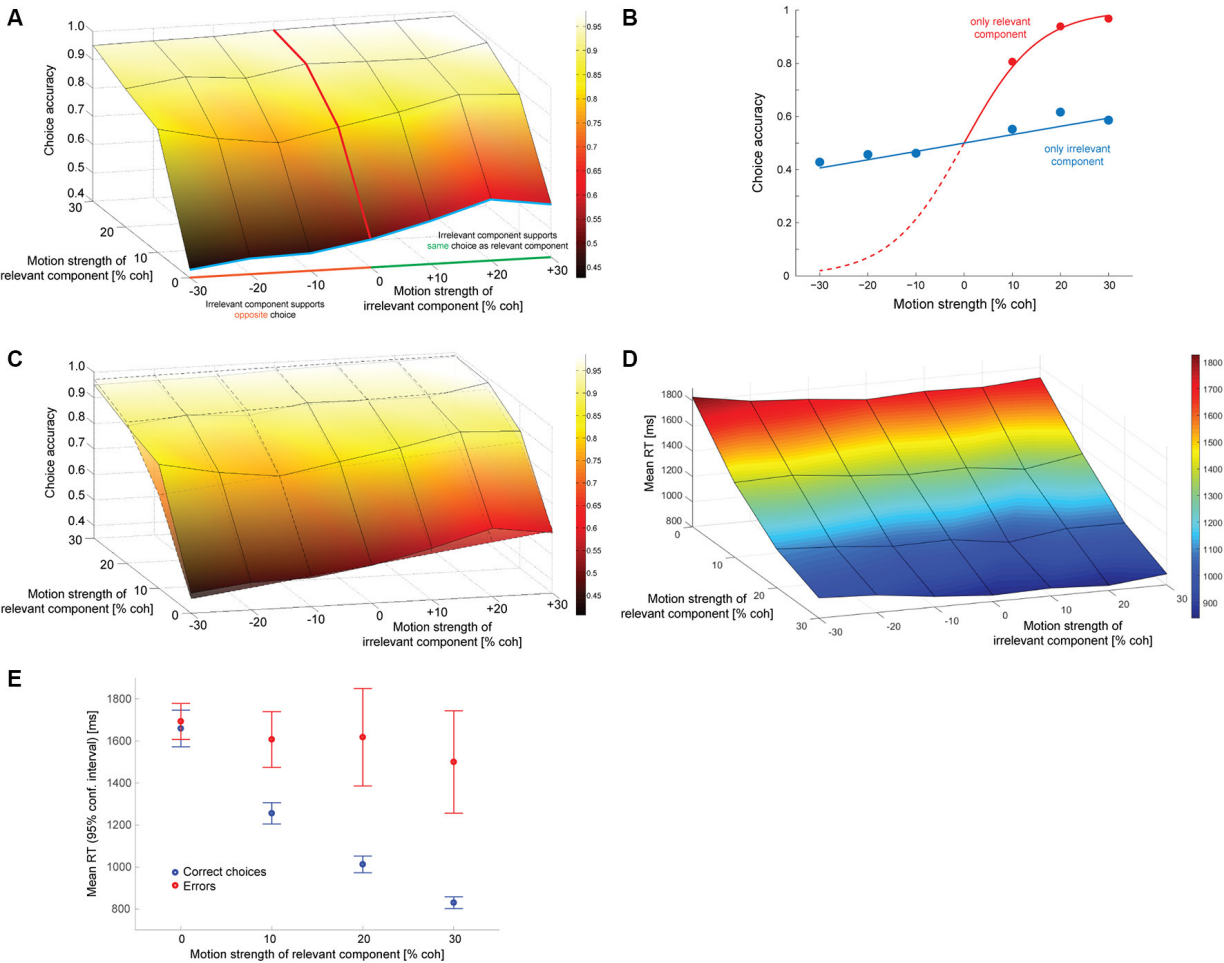
Decision behavior. **(A)** Choice accuracy (picking the target associated with the direction of motion along the relevant axis) as a function of the strength of the decision-relevant and irrelevant motion components. Positive values on the irrelevant motion strength axis indicate “congruent trials:” the irrelevant motion component provided evidence for choosing the same target as the relevant motion component (green part of axis). Negative values indicate “incongruent trials:” the irrelevant motion component provided evidence for choosing the opposite target (orange part of axis). The red line indicates trials with only relevant coherent motion, the blue line indicates trials with only irrelevant coherent motion. **(B)** Logistic fits to data from trials with either only relevant coherent motion [red; corresponds to red line in **(A)**] or only irrelevant coherent motion [blue; corresponds to blue line in **(A)**]. The filled circles reflect the data, lines are logistic fits based on maximum likelihood estimation. **(C)** Overlay of data (surface with solid lines) and a logistic function based on a linear combination of relevant and irrelevant motion strength (surface with dashed lines). The coefficients that have been used for constructing the surface are the ones derived from the fits in **(B)**. **(D)** Mean response times (RT) for all tested combinations of coherences. **(E)** Mean RTs of correct choices (blue) and errors (red) for trials without irrelevant coherent motion. Error bars reflect 95% confidence intervals.

Figure 2A shows how often subjects selected the correct target (the one associated with the direction of motion along the decision-relevant axis) as a function of the strength of the relevant and irrelevant motion signals. The decision-irrelevant motion component could either provide evidence for the same target as the relevant motion (“congruent trials”) or for the opposite target (“incongruent trials”). This is captured by the sign of the irrelevant motion strength: positive values indicate congruent trials, negative values incongruent trials. The choice is clearly dominated by the decision-relevant motion (steep slope of the psychometric function along the relevant motion axis), with accuracy increasing as the relevant motion strength increases. If subjects were able to ignore the irrelevant motion component completely the psychometric function would be flat along the irrelevant motion strength axis, which it is clearly not, indicating that the irrelevant sensory information had an impact on subjects' choices. We can quantify this by fitting a logistic function to the data from trials with only irrelevant coherent motion (blue line in Figure 2A) and estimating its slope, which is shown in Figure 2B (blue line). The estimated slope (α_*irr*_; see Data Analysis) is 1.26 (SE: 0.18), which is significantly different from zero (*p* < 10^−6^; *t*-test). A corresponding slope can also be estimated from trials with only relevant coherent motion (red lines in Figures 2A,B), which results in an α_*rel*_ of 13.0. The ratio of the two estimates, which is on the order of 10, provides a first estimate how much more impact the decision-relevant sensory information had on the choice compared to the irrelevant information. Thus, subjects were able to ignore the irrelevant information quite well, but not completely. The psychometric functions of individual subjects are shown in Supplementary Figure 2.

One possible explanation for the remaining effect of the decision-irrelevant sensory information on choice would be that the irrelevant information can only be suppressed to a certain degree and that some of the information always leaks through. The decision would then be based on a combination of the relevant sensory information and a weakened version of the irrelevant information. The simplest case of such a combination would be a linear superposition. Since the choice behavior in the basic version of the random-dot motion direction discrimination task is well-captured by a logistic function, we therefore wondered whether the choice behavior in our task could be captured by a logistic function based on a linear combination of decision-relevant and irrelevant motion strength. Figure 2C shows a superposition of the choice data (surface with solid lines) and such a logistic function (surface with dashed lines), using the two weights that had just been determined in Figure 2B. The general agreement is quite good, but if the decision were indeed based on such a linear combination we should be able to develop a model of the decision process that can account for all aspects of the behavioral data, including RTs. Figure 2D shows the mean RTs for all analyzed combinations of motion strengths (note that the direction of the relevant motion strength axis has been reversed compared to in Figures 2A,C to be able to look at the data surface). Variations in mean RT are dominated by the relevant motion strength, with responses to stimuli without relevant coherent motion being ~1 s slower than responses to stimuli with 30% coherent relevant motion. But mean RT is also affected by the strength of the irrelevant motion component: the psychometric function is again not flat along the irrelevant motion strength axis. Similar to previous data from the random-dot motion direction discrimination task, error RTs, on average, tended to be longer than RTs associated with correct responses. This is shown in Figure 2E for trials without irrelevant coherent motion. RT distributions will be shown later in **Figures 4**, **6** when comparing data and computational models.

### Computational model based on the idea of incomplete suppression of the irrelevant information

We developed a computational model based on the idea that decision-irrelevant sensory evidence is only incompletely suppressed. As a consequence, the decision is based on a linear combination of decision-relevant and irrelevant net sensory evidence. Consistent with a large body of previous work, the model is based on the integration-to-threshold framework, which can be formalized as a drift-diffusion model (Palmer et al., 2005; Ditterich, 2006; Niwa and Ditterich, 2008; Ratcliff and McKoon, 2008). In the case of our experiment the sensory evidence is represented by four pools of motion-sensitive neurons, tuned to the four cardinal directions. Since the multi-component random dot stimulus that has been used in this experiment is similar to the one that has been used in our earlier 3AFC study (Niwa and Ditterich, 2008), we took a similar approach to modeling the neural response to the stimulus. A more detailed justification can be found in Niwa and Ditterich (2008), but, briefly, the representation of the sensory evidence for a particular direction of motion was described as having a strong linear response to coherent motion in the preferred direction of the modeled pool of neurons and a weak linear response to non-coherent motion in the stimulus (random flickering; “noise dots”). This accounts for the roughly piecewise linear relationship between signed motion strength (positive motion strength corresponds to coherent motion in the preferred direction, negative values to motion in the null direction) and firing rate in the middle temporal area (MT) in response to random dot motion along a single axis (Britten et al., 1993). The typical direction tuning width of neurons in MT has been reported to be on the order of 40–50° (half-width at half-height) (Albright, 1984; Snowden et al., 1992; Treue et al., 2000). Motion orthogonal to the preferred direction is therefore not expected to cause considerable excitation, but we included a divisive normalization mechanism (Simoncelli and Heeger, 1998) to be able to account for interactions at the population level when multiple subpopulations of neurons that are tuned to different preferred directions are driven by simultaneously present components of coherent motion in multiple directions. The total amount of coherent motion in the stimulus is used as a proxy for the overall population response. Any potentially remaining small excitatory effect of orthogonal coherent motion on the response of the subpopulation of MT neurons providing evidence for a particular direction would not be direction-selective (due to the symmetry of direction tuning and both possible directions of motion having the same angular separation from the preferred direction) and would therefore be captured by our model by simply adjusting the strength of the normalization mechanism. The resulting equations can be found in Materials and Methods. The representation of sensory evidence is followed by an opponent readout stage. The difference between the activity of sensory neurons tuned to opposite directions of motion is taken as the net sensory evidence in favor of a particular direction. Net sensory evidence for each possible choice is then accumulated over time until a decision threshold is reached.

**Figure 3:**
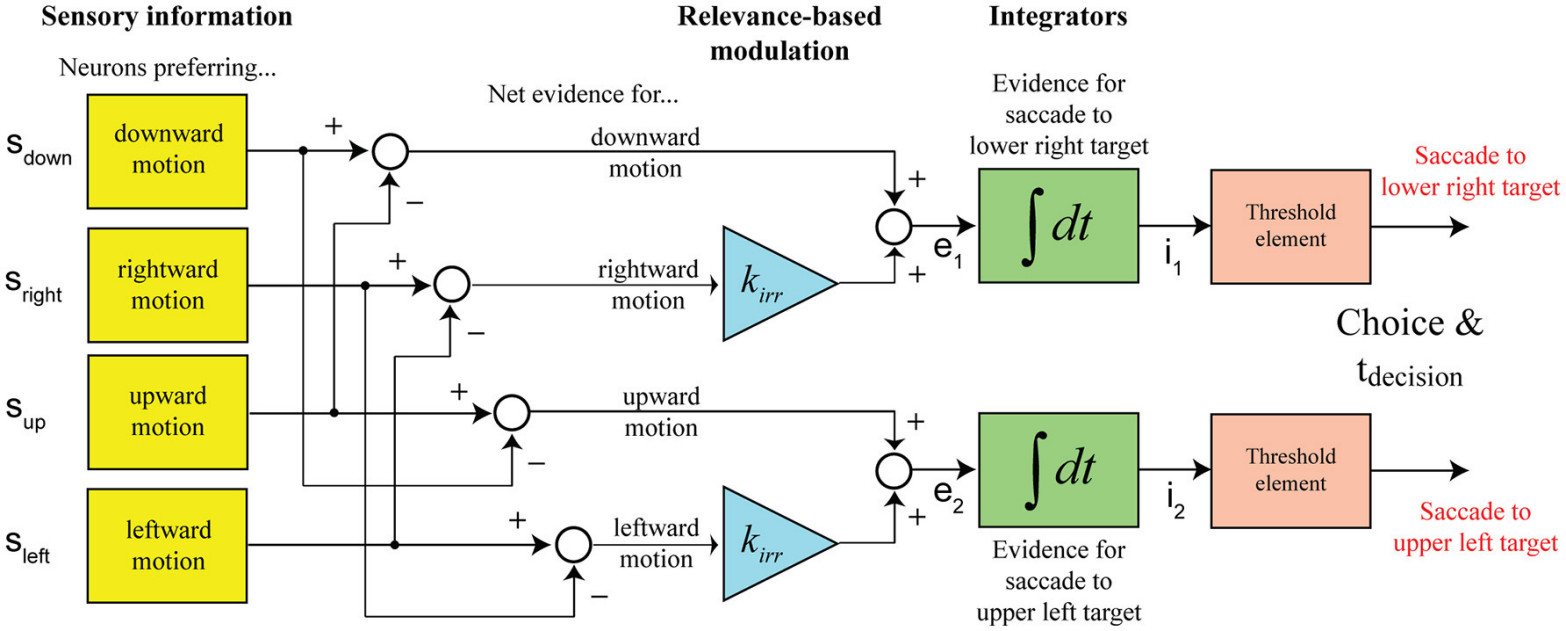
Structure of the computational model (shown for the example configuration in Figure 1). Decisions between the two possible action choices are based on accumulating net sensory evidence to a decision threshold. The key element of the model is that the net evidence signal that the decision is based on is a linear combination of decision-relevant (with a weight of 1) and irrelevant (with a weight of *k*_*irr*_) sensory information.

The critical addition to the model for being able to account for the results of our experiment is the relevance-based modulation stage. The net sensory evidence signal feeding into a decision integrator is a linear combination of the decision-relevant net sensory evidence along the cued motion axis and a damped version of the irrelevant net sensory evidence along the orthogonal axis. Figure 3 shows the structure of the proposed decision mechanism for the example configuration shown in Figure 1: targets are located to the top-left and bottom-right of the motion stimulus and the vertical axis is cued as decision-relevant. The race between the two shown integrators to the decision threshold is mathematically treated as a one-dimensional drift-diffusion process with an upper and a lower bound corresponding to the two possible choices. The model predicts a distribution of decision times. RTs were assumed to be the sum of the decision time and a randomized residual time, accounting for non-decision-related processing like response preparation and execution. Further details can be found in Materials and Methods.

**Figure 4:**
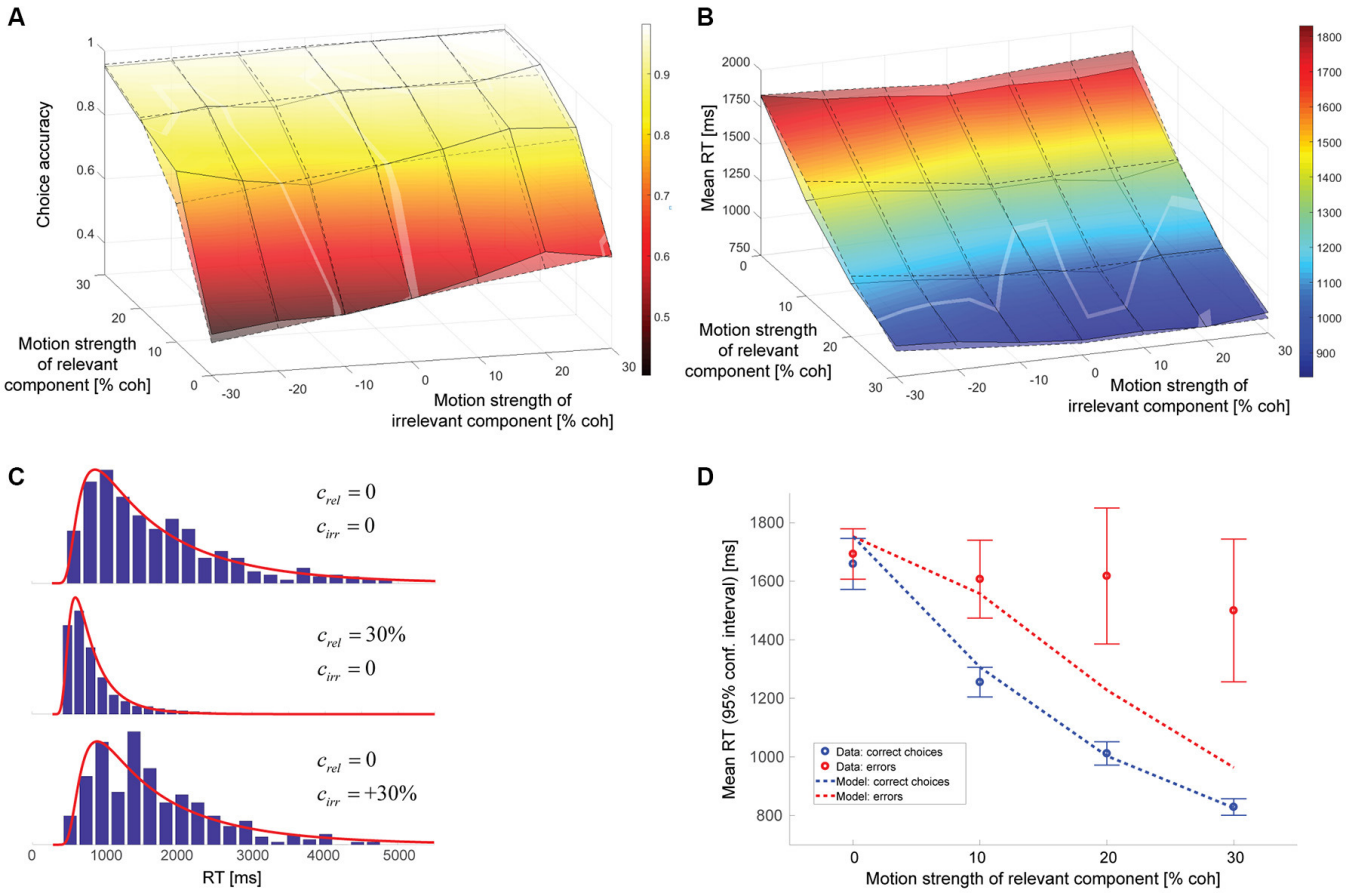
Model assuming incomplete suppression of irrelevant evidence. **(A)** Choice accuracy (surface with solid lines = data, surface with dashed lines = model). The solid lines connect the actual data points. The dashed lines connect the model predictions for the coherence combinations used in the experiment. The brighter, oddly-shaped structures in the surface plot are the result of the data and model surfaces intersecting. **(B)** Mean RT (surface with solid lines = data, surface with dashed lines = model). The solid lines connect the actual data points. The dashed lines connect the model predictions for the coherence combinations used in the experiment. The brighter, oddly-shaped structures in the surface plot are the result of the data and model surfaces intersecting. **(C)** Representative RT distributions for correct choices (blue histograms = data, red lines = model). Top: no coherent motion (441 trials); middle: only strong relevant coherent motion (852 trials); bottom: only strong irrelevant coherent motion (262 trials). The difference in number of available trials is a consequence of changes in accuracy with motion strength and trials with non-zero irrelevant motion strength being divided into congruent (positive signed irrelevant motion strength) and incongruent trials (negative signed irrelevant motion strength). **(D)** Mean RTs of correct choices (blue) and errors (red) for trials without irrelevant coherent motion. The circles reflect the data, the lines the model. Error bars indicate 95% confidence intervals.

The model parameters resulting from a maximum likelihood fit can be found in Table 1. The gain of the irrelevant information *k*_*irr*_ is estimated to be 0.084. Thus, according to this model, the decision-relevant net sensory evidence had 12 times more impact on the decision than the irrelevant information. See Discussion for a more general discussion of other model parameters. Figure 4 shows a comparison between experimental data and this model. As can be seen in Figure 4A, the psychometric function is well-accounted for. The surface with solid lines represents the data, the surface with dashed lines the model. The slope of the model psychometric function along the irrelevant motion strength axis is 1.30 (SE: 0.02), which is not significantly different from the value estimated from the data (*p* = 0.85). Figure 4B shows that mean RT is well-accounted for for the majority of motion strength combinations, but the model has a tendency to predict somewhat too long mean RTs when there is no relevant coherent motion. This is a point we will come back to later. Figure 4C shows three representative RT distributions: no coherent motion at the top, only strong relevant coherent motion in the middle, and only strong irrelevant coherent motion at the bottom. There is good agreement between the RT distribution shapes predicted by the model (red lines) and the actual distributions in the data (blue histograms). Due to the drift rate being allowed to show random variations across trials (see Materials and Methods), the model can account for the general observation that errors tended to be slower than correct choices (Ratcliff and Rouder, 1998), but still underestimates mean RTs on trials with strong relevant motion (Figure 4D).

**Table 1:** Model parameters and likelihoods.

**Parameters/values**	**Model using only linear superposition (leaking through of irrelevant information)**	**Model using only fraction of trials with opposite weighting (lapses in remembering cued component)**	**Model using only fraction of trials (fixed) with opposite weighting**	**Model using linear superposition and fraction of trials with opposite weighting**
**Sensory gain** *g* (to obtain drift rate in *ms*^−1^)	0.00780	0.00894	0.0103	0.00884
**Response to noise dots** *k*_*n*_	0.0433	0.0893	0.0815	0.0853
**Strength of divisive normalization** *k*_*s*_	0.396	0.694	1.08	0.655
**SD of drift rate** σ_*DR*_ [*ms*^−1^]	5.68·10^−4^	5.28·10^−4^	4.78·10^−4^	5.47·10^−4^
**Mean-dependent variability of sensory response** *k*_*v*_	0.235	0.245	0.223	0.261
**Stimulus-independent noise** $\sigma_{n}^{2}$ [*ms*^−1^]	4.17·10^−4^	2.41·10^−4^	2.56·10^−4^	2.33·10^−4^
**Gain of irrelevant sensory information** *k*_*irr*_	0.0844	**0** (enforced)	**0** (enforced)	0.0647
**Proportion of trials with opposite weighting** *p*_*wrong*_*axis*_	**0** (enforced)	0.0421	**0.16** (enforced)	0.0306
**Mean residual time** $\bar{t_{res}}$ [ms]	347	346	345	350
**SD of residual time** σ_*t*_*res*__ [ms]	37.5	37.2	39.9	40.0
**Log likelihood**	−113,082	−113,088	−113,332 (worst fit)	−113,046 (best fit)
**Slope of psychometric function for trials with only irrelevant coherent motion** α_*model*_	1.30 (SE: 0.02) (determined after model fit)	0.32 (SE: 0.11) (determined after model fit)	1.28 (SE: 0.19) (determined after model fit)	1.32 (SE: 0.10) (determined after model fit)

*Model parameters shown in **bold face** were enforced; model parameters shown in normal font were obtained through maximum likelihood estimation. The slope of the psychometric function (last row) is not an additional model parameter and was determined after the model fit*.

### Computational model based on the idea that subjects sometimes forget which component has been cued as relevant

**Figure 5:**
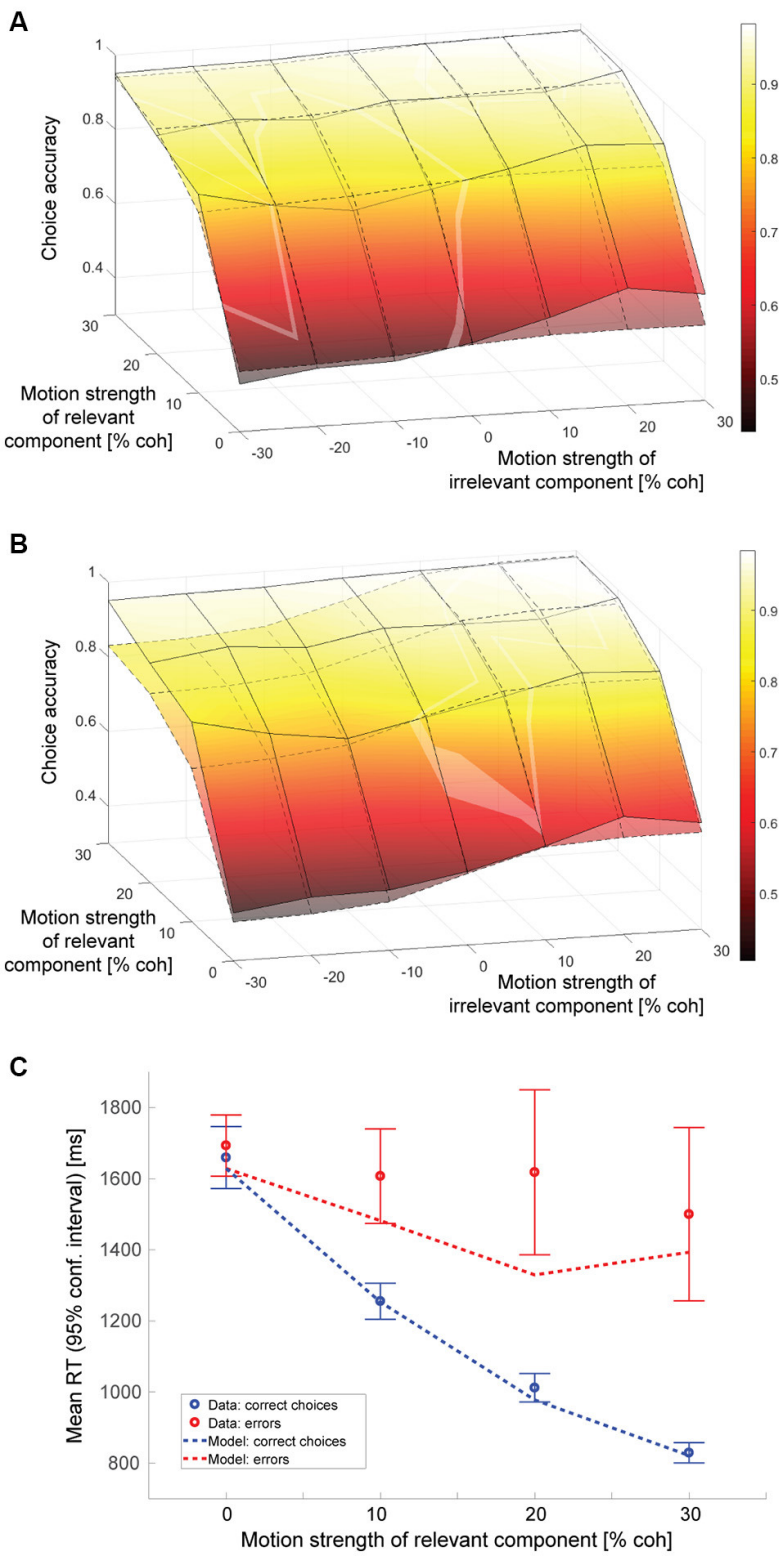
Model assuming cue forgetting. **(A)** Choice accuracy (surface with solid lines = data, surface with dashed lines = model; see Figure 4A for additional information). All model parameters were determined using maximum likelihood estimation. Note that the model psychometric function has a much shallower slope along the irrelevant motion strength axis. **(B)** Proportion of trials on which the decision was based on the wrong motion component was fixed at 16%; remaining model parameters determined using maximum likelihood estimation. The slopes of the psychometric function along the irrelevant motion strength axis now match, but the model predicts substantially lower accuracy on incongruent trials with strong relevant motion than observed in the data. **(C)** Mean RTs of correct choices and errors (see Figure 4D for additional information).

An alternative explanation for the decisions being affected by the irrelevant sensory information would be that subjects sometimes, on a certain fraction of the trials, have forgotten which motion axis had been cued and end up making a decision based on the motion along the axis that had not been cued as decision-relevant. In this scenario, trials would be a mixture of a majority of trials where the decision is based on the relevant stimulus component and a minority of trials where the decision is based on the irrelevant stimulus component. The resulting psychometric function would show an effect of the irrelevant motion strength even if subjects were able to suppress the irrelevant information completely on those trials where the appropriate motion component was used. Could this idea potentially also explain the collected data? Fitting a model with complete suppression of the irrelevant net sensory evidence on the majority of trials and assuming that the decision is purely based on the irrelevant net sensory evidence on a certain fraction of trials results in the psychometric function shown in Figure 5A. The surface with solid lines represents the data, the surface with dashed lines the model. The model parameters can be found in Table 1. The maximum likelihood estimate of how often a decision would have to be based on the wrong component is on the order of 4%. The slope of the model psychometric function along the irrelevant motion strength axis is, however, only 0.32 (SE: 0.11), which is approximately one quarter of the value estimated from the data and significantly smaller (*p* = 10^−5^). The model therefore cannot account for the magnitude of the effect that decision-irrelevant sensory information had on choice. To match the impact one would have to postulate that subjects based their decision on the wrong component of the stimulus in ~16% of the trials. This results in a slope of the model psychometric function along the irrelevant motion strength axis of 1.28 (SE: 0.19), which is not significantly different from the value estimated from the data (*p* = 0.96). All model parameters can be found in Table 1. The predicted psychometric function is shown by the surface with dashed lines in Figure 5B. The model now predicts substantially lower accuracy on incongruent trials with strong relevant motion as observed in the experiment and therefore again cannot account for the data.

Despite this model's failure to capture the impact of the irrelevant sensory information on choice behavior, the likelihood associated with the model fit was not much worse than the one associated with fitting the previously discussed linear combination model, suggesting that there was some other aspect of the dataset that could be explained by the forgetting model, but not the linear combination model. As can be seen in Figure 5C, the forgetting model can account for longer error RTs, even when the relevant motion is strong. Slow errors on trials with high relevant coherence are very unlikely according to the linear combination model. They are more likely, however, according to the model based on cue forgetting as they could have been the consequence of basing the decision on the wrong motion component, which can have low coherence even when the cued component is strong. We therefore fitted a final, hybrid model, assuming that decision-irrelevant sensory information would never be fully suppressed and that subjects would sometimes forget the cue and therefore end up basing their decision on the wrong motion component on a fraction of the trials.

### Hybrid model with incomplete suppression of irrelevant evidence and cue forgetting

**Figure 6:**
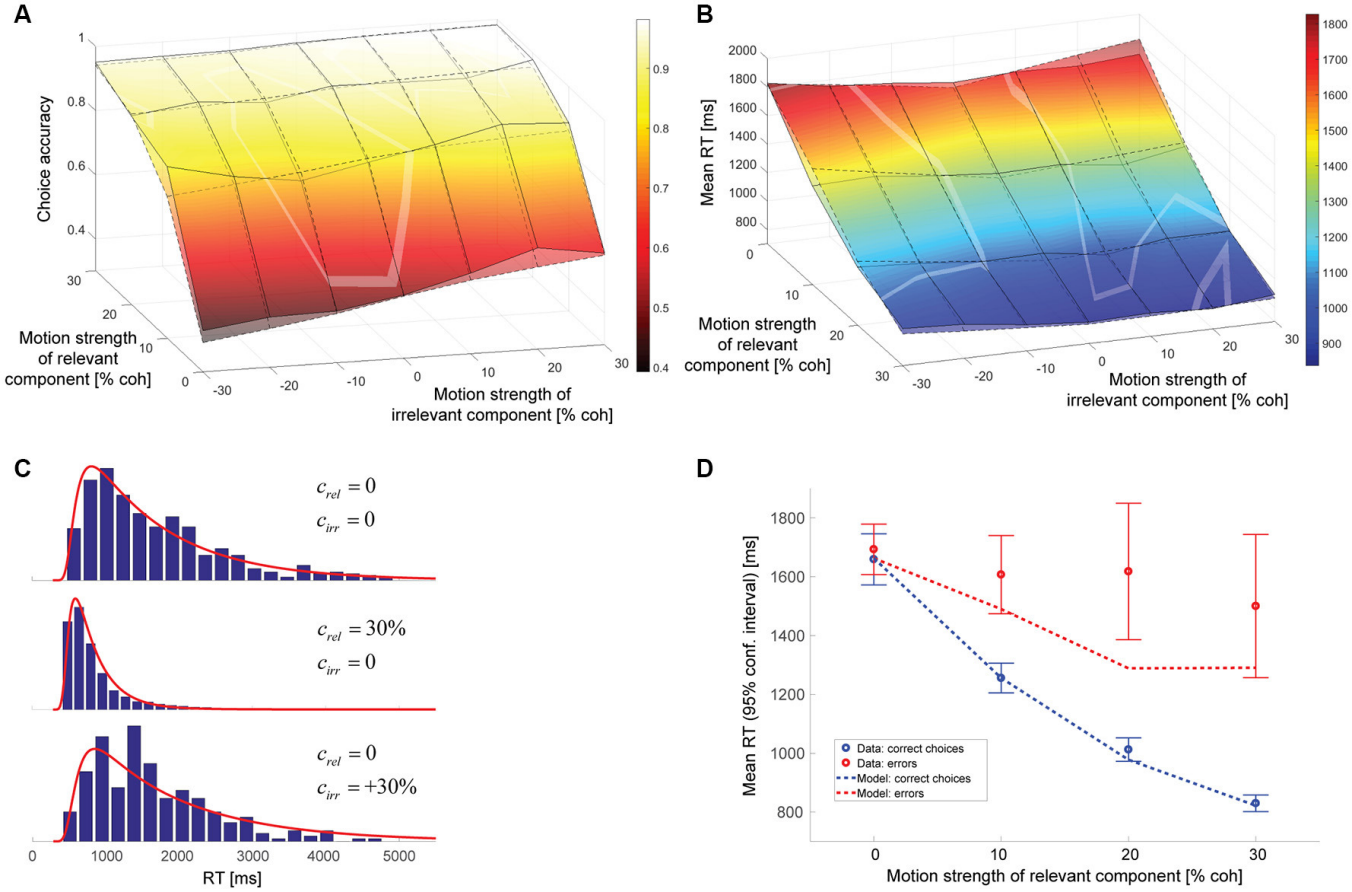
Hybrid model combining incomplete suppression of irrelevant evidence and cue forgetting. **(A)** Choice accuracy (surface with solid lines = data, surface with dashed lines = model; see Figure 4A for additional information). **(B)** Mean RT (surface with solid lines = data, surface with dashed lines = model; see Figure 4B for additional information). **(C)**, RT distributions (blue histograms = data, red lines = model; see Figure 4C for additional information). **(D)**, Mean RTs of correct choices and errors (see Figure 4D for additional information).

The parameters of the hybrid model can again be found in Table 1. The data is best accounted for with a gain of the decision-irrelevant net sensory evidence *k*_*irr*_ of 0.065, corresponding to the decision-relevant information having 15 times more impact on the decision than the irrelevant information, and the decision being based on the wrong motion component in 3.1% of the trials. Figure 6 shows that the psychometric function (Figure 6A), mean RTs (Figure 6B), and RT distributions (Figure 6C) are well-captured by this model. Error RTs are still somewhat underestimated, but mostly within the 95% confidence intervals provided by the data (Figure 6D). The slope of the model psychometric function along the irrelevant motion strength axis is 1.32 (SE: 0.10), which is not significantly different from the value estimated from the data (*p* = 0.80). Also note that the match between data and model in mean RT for trials without relevant coherent motion is improved over Figure 4B.

## Discussion

We have asked human subjects to make perceptual decisions in the presence of both decision-relevant and irrelevant sensory information. While the decisions were mostly based on the relevant information, the irrelevant information still had a clearly measurable influence. We have demonstrated that this effect cannot purely be the consequence of subjects sometimes forgetting which component of the sensory information had been cued as decision-relevant. Instead, one has to postulate that the decision mechanism is unable to completely suppress the irrelevant evidence such that some of the information leaks through and ends up affecting the decision. Using different models, we have estimated the strength of the remaining irrelevant sensory evidence to be between 6 and 10% of the strength of the relevant signal. The selection mechanism is mathematically well-described by a linear gain modulation such that the decision can be described as being based on a linear combination of decision-relevant and irrelevant net sensory evidence.

### Model parameters and their interpretation

The ratio between the variance of the sensory response and its mean was estimated to be between 0.24 and 0.26 (excluding the model with an enforced proportion of trials being dominated by the wrong stimulus component). This is very similar to our previous estimate of 0.26 in a study on multi-alternative perceptual decision-making (Niwa and Ditterich, 2008). This ratio is clearly sub-Poisson, which is expected if the evidence for motion in a particular direction is not provided by a single neuron, but rather a population of neurons. On the other hand, it is clearly not negligible, which can be due to a limited size of the population and/or positive correlations between neurons in the population, which have, for example, been reported in area MT (Zohary et al., 1994). In addition to fluctuations of the decision signal that scale with the activity of neurons representing the sensory evidence, we also fitted a stimulus-independent component of the variance of the decision signal, which was estimated to be between 2·10^−4^ and 4·10^−4^ *ms*^−1^. This roughly corresponds to the amount of noise contributed by a pool of neurons driven by 15%-coherent motion in the preferred direction. The overall amount of noise on the decision signal is therefore dominated by the fixed component in the case of weak stimuli, but the stimulus-dependent component can exceed the fixed component in the presence of strong stimuli.

The mean residual time was consistently estimated to be about 350 ms, which is similar to the value obtained in Niwa and Ditterich (2008). Mean decision times were therefore ranging from ~500 to 1,400 ms across conditions, clearly dominating mean RT. Similarly, the standard deviation of the residual time was consistently estimated to be on the order of 40 ms. Across-trial variability in RT was therefore also clearly dominated by variability in decision time.

### Potential contributions by feature-based attention

Decision-relevant and irrelevant sensory information were presented at the same spatial location in our task. Spatial attention could therefore not play a role in separating decision-relevant from irrelevant evidence. Feature-based attention, however, could have contributed: paying attention to a particular axis of motion might modulate neural activity in such a way that responses to motion along this axis are enhanced, whereas responses to motion along the orthogonal axis might be reduced. Treue and colleagues have reported changes in the firing rate of MT neurons in response to a motion stimulus inside the receptive field depending on the direction of a second, attended motion stimulus outside the receptive field (Treue and Martinez Trujillo, 1999; Martinez-Trujillo and Treue, 2004; Maunsell and Treue, 2006). While robust, reported changes in firing rate due to feature-based attention are of limited size, with firing rate ratios typically much smaller than two. Since in our task the firing rate of MT neurons is roughly proportional to the motion strength, reducing the effectiveness of the irrelevant motion component by a factor of at least 10, as determined in this study, by modulating the firing rate of sensory neurons would require a rate modulation of similar size, which is about an order of magnitude larger than the effects reported in the literature. Firing rate modulations in sensory representations due to feature-based attention are therefore not expected to be sufficient to separate decision-relevant from irrelevant information.

Sasaki and Uka trained monkeys to make judgments about either the direction of motion or about stereoscopic depth of 3D random dot patterns (Sasaki and Uka, 2009). Recordings from area MT revealed that the firing rate of neurons was essentially unaffected by whether the monkeys had to make a decision about the direction of motion or about depth. This observation is supported by preliminary results from our own lab. We recorded from area MT while a monkey performed the same task as the human subjects in the reported study here and also found no substantial changes in firing rate depending on whether the recorded neuron carried decision-relevant or irrelevant motion information on any given trial (Ditterich, 2015). The observations from both labs suggest that the separation of decision-relevant from irrelevant information is not achieved by modulating the firing rate of neurons in sensory representations. The single-unit data does not rule out that parameters of the population response other than the firing rate could be modulated by decision-relevance. For example, Cohen and Newsome reported changes in the correlation structure of the neural response depending on whether pairs of neurons provided evidence for the same choice or for opposite choices (Cohen and Newsome, 2008). Future studies will have to show whether such changes could contribute to the separation of decision-relevant from irrelevant sensory information.

### Neural implementation of the selection mechanism

Mitani et al. did a modeling study of the already mentioned Sasaki and Uka dataset (Mitani et al., 2013). Similar to our conclusion, they determined that the primary reason for choices being affected by decision-irrelevant sensory information was “interference,” i.e., task-irrelevant sensory evidence not being fully suppressed, rather than “task misapplication,” which corresponds to making a decision about the wrong axis of motion in our task. In their case, the argument was based on the addition of “task misapplication” to the model not leading to a substantial improvement of the model fit. We saw an improvement, but demonstrated that “task misapplication” (using the wrong component) alone would lead to a data pattern that is incompatible with the observed choice data, whereas leaking through of the irrelevant information alone predicts a pattern that is compatible with the observed choice data. Mitani et al. considered a “gated-integrator” model, which is roughly equivalent to the type of model considered here, and found that it was able to account for the choice behavior (decision times were not measured in their case). This model, however, was not able to account for the choice probability (CP) time course of recorded MT neurons. The authors therefore favored a “double-leaky-integrator” model: decision-relevant and irrelevant sensory evidence are assumed to be accumulated in separate integrators, with the task-irrelevant integrator being much leakier than the relevant one. There is, however, an alternative explanation for the reported late CP effect as put forward by Nienborg and Cumming (2010). It could be the consequence of a choice-related feedback signal rather than a feedforward phenomenon, which would restore compatibility of the data with a mechanism that is based on integrating a linear combination of decision-relevant and irrelevant sensory information.

Mante and colleagues trained monkeys to make a judgment about either the direction of motion or the color content of colored random dot stimuli (Mante et al., 2013). Single-unit recordings from prefrontal cortex indicated that individual neurons carried mixtures of decision-related signals. The authors determined that it was possible to decode a number of signals from the population response: unmodulated decision-relevant as well as irrelevant sensory evidence and an accumulated version of mostly the relevant sensory evidence. Inspired by this experimental observation, the authors developed a neural network model of selective integration, which could explain how decisions could almost exclusively be based on the relevant sensory evidence as well as the types of neural signals observed in prefrontal cortex. Effectively, although the irrelevant momentary evidence signal itself does not get attenuated in this model, it acts like a gated integrator or gain modulator depending on task relevance, as the irrelevant momentary evidence does not move the state of the system along the decision axis, which means that this component of the sensory evidence is not being integrated. This study suggests that the problem of selective decision-making might completely be solved by a local circuit in prefrontal cortex.

Siegel and colleagues recorded multi-unit activity from a variety of cortical areas while monkeys also performed a color vs. motion discrimination task (Siegel et al., 2015). The authors reported that various task-related signals were present in large, distributed networks of brain areas. Future studies will therefore have to show where and how critical contributions to the separation of decision-relevant from irrelevant sensory information are made in the brain. Our results provide computational constraints that will have to be met by candidate neural mechanisms.

## Author contributions

JD conceived the study, analyzed the data, developed the computational models, and wrote most of the manuscript. UA, CM, and BO collected the data and contributed to writing the manuscript.

### Conflict of interest statement

The authors declare that the research was conducted in the absence of any commercial or financial relationships that could be construed as a potential conflict of interest.

## References

[B1] Albright T. D. (1984). Direction and orientation selectivity of neurons in visual area MT of the macaque. J. Neurophysiol. 52, 1106–1130. 6520628 10.1152/jn.1984.52.6.1106

[B2] Brainard D. H. (1997). The psychophysics toolbox. Spat. Vis. 10, 433–436. 10.1163/156856897X00357 9176952

[B3] Britten K. H. Shadlen M. N. Newsome W. T. Movshon J. A. (1993). Responses of neurons in macaque MT to stochastic motion signals. Vis. Neurosci. 10, 1157–1169. 10.1017/S0952523800010269 8257671

[B4] Cohen M. R. Newsome W. T. (2008). Context-dependent changes in functional circuitry in visual area MT. Neuron 60, 162–173. 10.1016/j.neuron.2008.08.007 18940596 PMC2652654

[B5] Davies D. L. Bouldin D. W. (1979). A cluster separation measure. IEEE Trans. Pattern Anal. Mach. Intell. 1, 224–227. 10.1109/TPAMI.1979.4766909 21868852

[B6] Ditterich J. (2006). Stochastic models of decisions about motion direction: behavior and physiology. Neural Netw. 19, 981–1012. 10.1016/j.neunet.2006.05.042 16952441

[B7] Ditterich J. (2015). Where and how are relevant sensory signals for perceptual decisions selected in the brain and mapped onto appropriate actions?, in Program No. 722.06. 2015 Neuroscience Meeting Planner (Chicago, IL: Society for Neuroscience).

[B8] Mante V. Sussillo D. Shenoy K. V. Newsome W. T. (2013). Context-dependent computation by recurrent dynamics in prefrontal cortex. Nature 503, 78–84. 10.1038/nature12742 24201281 PMC4121670

[B9] Martinez-Trujillo J. C. Treue S. (2004). Feature-based attention increases the selectivity of population responses in primate visual cortex. Curr. Biol. 14, 744–751. 10.1016/j.cub.2004.04.028 15120065

[B10] Maunsell J. H. Treue S. (2006). Feature-based attention in visual cortex. Trends Neurosci. 29, 317–322. 10.1016/j.tins.2006.04.001 16697058

[B11] Meeker W. Q. Escobar L. A. (1998). Statistical Methods for Reliability Data. New York, NY: Wiley.

[B12] Mitani A. Sasaki R. Oizumi M. Uka T. (2013). A leaky-integrator model as a control mechanism underlying flexible decision making during task switching. PLoS ONE 8:e59670. 10.1371/journal.pone.0059670 23533641 PMC3606137

[B13] Nienborg H. Cumming B. (2010). Correlations between the activity of sensory neurons and behavior: how much do they tell us about a neuron's causality? Curr. Opin. Neurobiol. 20, 376–381. 10.1016/j.conb.2010.05.002 20545019 PMC2952283

[B14] Niwa M. Ditterich J. (2008). Perceptual decisions between multiple directions of visual motion. J. Neurosci. 28, 4435–4445. 10.1523/JNEUROSCI.5564-07.2008 18434522 PMC6670944

[B15] Palmer J. Huk A. C. Shadlen M. N. (2005). The effect of stimulus strength on the speed and accuracy of a perceptual decision. J. Vis. 5, 376–404. 10.1167/5.5.1 16097871

[B16] Pelli D. G. (1997). The VideoToolbox software for visual psychophysics: transforming numbers into movies. Spat. Vis. 10, 437–442. 10.1163/156856897X00366 9176953

[B17] Ratcliff R. (1979). Group reaction time distributions and an analysis of distribution statistics. Psychol. Bull. 86, 446–461. 10.1037/0033-2909.86.3.446 451109

[B18] Ratcliff R. McKoon G. (2008). The diffusion decision model: theory and data for two-choice decision tasks. Neural Comput. 20, 873–922. 10.1162/neco.2008.12-06-420 18085991 PMC2474742

[B19] Ratcliff R. Rouder J. N. (1998). Modeling response times for two-choice decisions. Psychol. Sci. 9, 347–356. 10.1111/1467-9280.00067

[B20] Roitman J. D. Shadlen M. N. (2002). Response of neurons in the lateral intraparietal area during a combined visual discrimination reaction time task. J. Neurosci. 22, 9475–9489. 12417672 10.1523/JNEUROSCI.22-21-09475.2002 PMC6758024

[B21] Rousseeuw P. J. (1987). Silhouettes - a graphical aid to the interpretation and validation of cluster-analysis. J. Comput. Appl. Math. 20, 53–65. 10.1016/0377-0427(87)90125-7

[B22] Sapir A. D'avossa G. Mcavoy M. Shulman G. L. Corbetta M. (2005). Brain signals for spatial attention predict performance in a motion discrimination task. Proc. Natl. Acad. Sci. U.S.A. 102, 17810–17815. 10.1073/pnas.0504678102 16306268 PMC1308888

[B23] Sasaki R. Uka T. (2009). Dynamic readout of behaviorally relevant signals from area MT during task switching. Neuron 62, 147–157. 10.1016/j.neuron.2009.02.019 19376074

[B24] Shadlen M. N. Newsome W. T. (2001). Neural basis of a perceptual decision in the parietal cortex (area LIP) of the rhesus monkey. J. Neurophysiol. 86, 1916–1936. 11600651 10.1152/jn.2001.86.4.1916

[B25] Siegel M. Buschman T. J. Miller E. K. (2015). Cortical information flow during flexible sensorimotor decisions. Science 348, 1352–1355. 10.1126/science.aab0551 26089513 PMC4721574

[B26] Simoncelli E. P. Heeger D. J. (1998). A model of neuronal responses in visual area MT. Vision Res. 38, 743–761. 10.1016/S0042-6989(97)00183-1 9604103

[B27] Smith P. L. (2000). Stochastic dynamic models of response time and accuracy: a foundational primer. J. Math. Psychol. 44, 408–463. 10.1006/jmps.1999.1260 10973778

[B28] Snowden R. J. Treue S. Andersen R. A. (1992). The response of neurons in areas V1 and MT of the alert rhesus monkey to moving random dot patterns. Exp. Brain Res. 88, 389–400. 10.1007/BF02259114 1577111

[B29] Treue S. Martinez Trujillo J. C. (1999). Feature-based attention influences motion processing gain in macaque visual cortex. Nature 399, 575–579. 10.1038/21176 10376597

[B30] Treue S. Hol K. Rauber H. J. (2000). Seeing multiple directions of motion-physiology and psychophysics. Nat. Neurosci. 3, 270–276. 10.1038/72985 10700260

[B31] Wendelken C. Ditterich J. Bunge S. A. Carter C. S. (2009). Stimulus and response conflict processing during perceptual decision making. Cogn. Affect. Behav. Neurosci. 9, 434–447. 10.3758/CABN.9.4.434 19897796

[B32] Wilimzig C. Tsuchiya N. Fahle M. Einhauser W. Koch C. (2008). Spatial attention increases performance but not subjective confidence in a discrimination task. J. Vis. 8, 7–10. 10.1167/8.5.7 18842078

[B33] Wyart V. Myers N. E. Summerfield C. (2015). Neural mechanisms of human perceptual choice under focused and divided attention. J. Neurosci. 35, 3485–3498. 10.1523/JNEUROSCI.3276-14.2015 25716848 PMC4402727

[B34] Zizlsperger L. Sauvigny T. Haarmeier T. (2012). Selective attention increases choice certainty in human decision making. PLoS ONE 7:e41136. 10.1371/journal.pone.0041136 22815942 PMC3397971

[B35] Zohary E. Shadlen M. N. Newsome W. T. (1994). Correlated neuronal discharge rate and its implications for psychophysical performance. Nature 370, 140–143. 10.1038/370140a0 8022482

