# Supplementary material for: Perceptual Decisions in the Presence of Relevant and Irrelevant Sensory Evidence
Source: Front Neurosci. 2017 Nov 10;11:618. doi: 10.3389/fnins.2017.00618 (PMC5686122; doi:10.3389/fnins.2017.00618)
Supplement: Supplementary file 1 [file Image1.pdf]

Supplementary Material

Perceptual Decisions in the Presence of Relevant and Irrelevant Sensory Evidence

Ursula M. Anders, Charlotte S. McLean, Bowen Ouyang, Jochen Ditterich*

*** Correspondence:** Jochen Ditterich: jditterich@ucdavis.edu

1 Supplementary Figures and Tables

1.1 Supplementary Figures

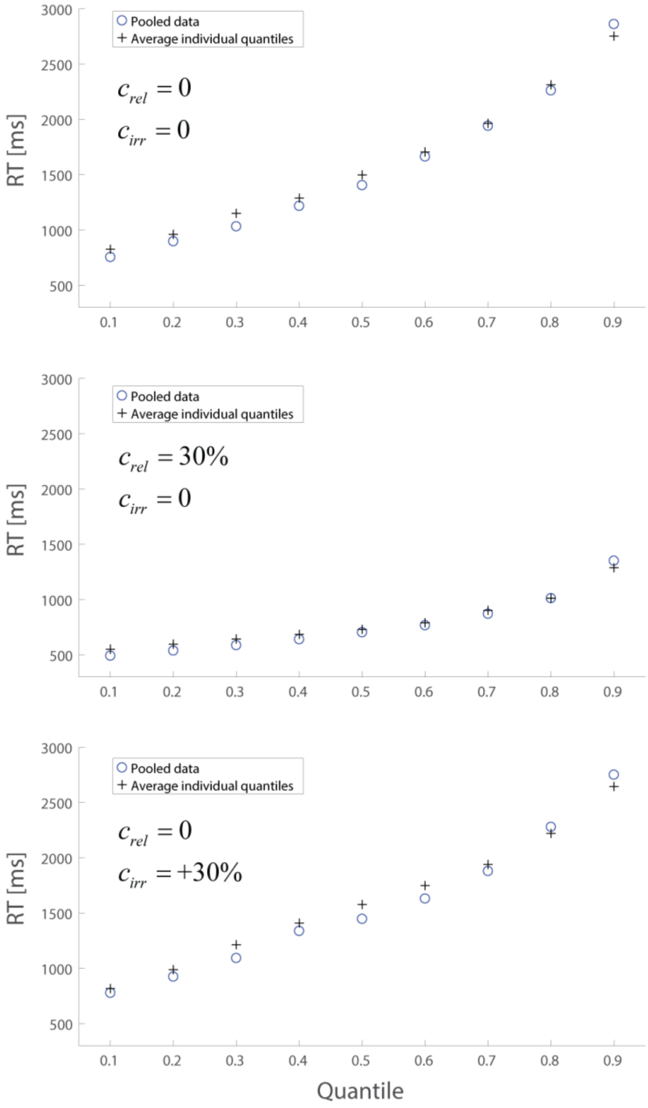

Supplementary Figure 1. Comparison between RT distributions obtained from the pooled data (blue circles) and group RT distributions (black plusses), which were obtained by determining the quantiles of individual subjects' RT distributions and averaging those across subjects (Ratcliff, 1979). The RT distributions are shown for correct choices in response to three different combinations of relevant and irrelevant motion strength.

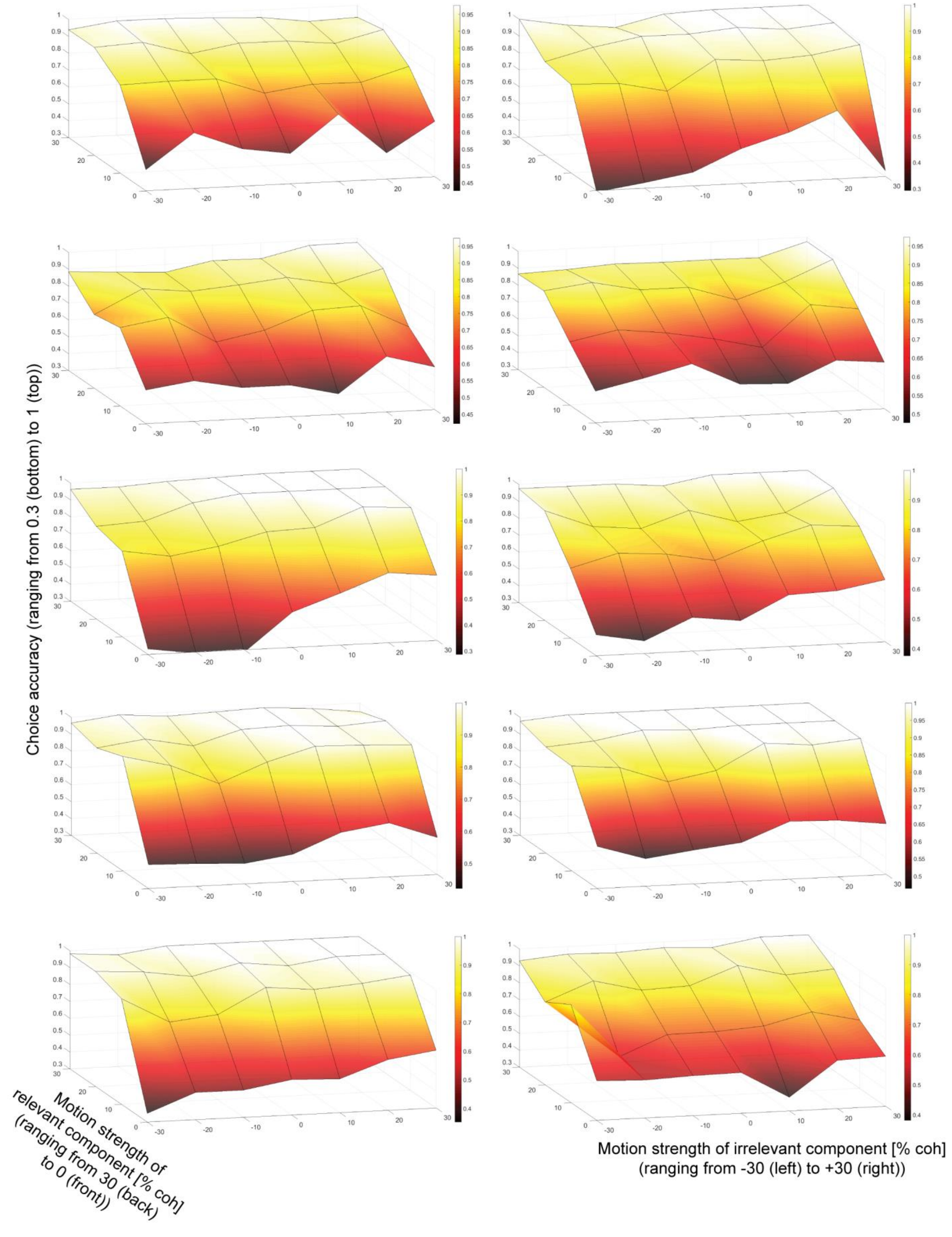

Supplementary Figure 2. Individual psychometric functions. Format is the same as in Fig. 2A, only the range of the choice accuracy axis has been increased to now start at 0.3.

2 References

Ratcliff, R. (1979). Group reaction time distributions and an analysis of distribution statistics. *Psychol Bull* 86**,** 446-461.
